# A Biomechanical Study of Pull-Out Strength in Fenestrated Pedicle Screws Inserted Into Osteoporotic Cancellous Bone Models Using the Jamshidi Needle: A Comparative Analysis of Cement Volumes and Augmentation Techniques

**DOI:** 10.7759/cureus.104976

**Published:** 2026-03-10

**Authors:** Roshyn S Khosa, Mohd Hezery Bin Harun, Teck Siang Lim, Shukri Safian, Mohd Na'im Abdullah

**Affiliations:** 1 Orthopaedics and Traumatology, Universiti Putra Malaysia, Serdang, MYS; 2 Aerospace Engineering, Universiti Putra Malaysia, Serdang, MYS

**Keywords:** biomechanics, cement augmentation, fenestrated pedicle screws, jamshidi needle technique, low-volume cement, osteoporotic bone model, pedicle screw, pull-out strength, spinal instrumentation stability

## Abstract

Background

Osteoporosis is increasingly prevalent in aging populations and remains a major barrier to achieving reliable spinal fixation. Poor cancellous bone quality compromises the bone-implant interface, leading to higher rates of pedicle screw loosening, construct failure, and revision surgery. Cement augmentation improves fixation strength but is associated with volume-dependent complication risk, creating a clinical need for low-volume, mechanically efficient augmentation strategies. Concurrently, modern minimally invasive fixation increasingly relies on Jamshidi needle-guided tract preparation, which may influence cancellous bone compaction and cement containment. Despite these evolving surgical workflows, limited biomechanical data exist evaluating the combined effects of cement delivery technique and low-dose cement volume in Jamshidi-guided screw insertion. This study investigated whether the cement delivery technique exerts a greater biomechanical influence than cement volume when using fenestrated pedicle screws.

Methods

This in vitro biomechanical comparative study utilized standardized osteoporotic cancellous polyurethane foam blocks. Fenestrated cannulated pedicle screws (6.5 × 40 mm) were inserted using a Jamshidi needle and guidewire technique. Five experimental groups were evaluated (n = 3 per group): Group A (no cement), Group B (0.5 mL cannulated injection), Group C (1.0 mL cannulated injection), Group D (0.5 mL prefill), and Group E (1.0 mL prefill). After cement polymerization, axial pull-out testing was performed using a universal testing machine. Primary outcomes included pull-out strength, axial stiffness, and displacement to failure. Statistical analysis was conducted using the Kruskal-Wallis test with post hoc pairwise comparisons.

Results

Significant overall differences were observed across experimental groups for all biomechanical parameters (all p = 0.01). Mean pull-out strength increased from 524.42 ± 12.40 N in the control group to 874.05 ± 17.41 N with 0.5 mL cannulated injection and 1030.32 ± 37.05 N with 1.0 mL injection, representing improvements of 66.7% and 96.5%, respectively. Prefill augmentation produced smaller gains, with 595.02 ± 41.74 N (+13.5%) for 0.5 mL and 760.08 ± 39.37 N (+44.9%) for 1.0 mL. Axial stiffness increased from 230.35 ± 1.54 N/mm in controls to 324.15 ± 5.57 N/mm and 336.10 ± 6.04 N/mm in injection groups, while prefill augmentation resulted in more modest improvements. Displacement to failure was highest in injection groups (2.70 ± 0.05 mm and 3.07 ± 0.14 mm), compared with the control (2.28 ± 0.06 mm) and prefill groups. Notably, 0.5 mL cannulated injection consistently outperformed 1.0 mL prefill augmentation across primary biomechanical outcomes.

Conclusions

In Jamshidi-guided insertion of fenestrated pedicle screws, the cement delivery technique has a greater biomechanical impact than low-dose cement volume. Cannulated cement injection provides superior fixation stability, even at reduced cement volumes, whereas low-volume prefill augmentation offers limited mechanical benefit. These findings support a technique-driven, low-volume cement augmentation strategy for optimizing pedicle screw fixation in osteoporotic bone while potentially reducing cement-related complications.

## Introduction

Pedicle screw instrumentation remains a cornerstone of posterior spinal stabilization; however, achieving durable fixation in osteoporotic bone continues to represent a major biomechanical challenge. Reduced trabecular bone density, thinning of cancellous struts, and impaired load-sharing capacity weaken the screw-bone interface, resulting in higher rates of loosening, construct failure, and revision surgery compared with those in patients with normal bone quality [1,2].

Biomechanical investigations have demonstrated that the quantity of bone cement significantly influences pedicle screw anchorage in osteoporotic vertebrae [3]. As the global population ages and the prevalence of osteoporosis increases [4], the demand for reliable fixation strategies in poor-quality bone has become increasingly important in modern spine surgery. Furthermore, the mechanical performance of pedicle screws is closely associated with bone mineral density, with reduced density associated with diminished pull-out strength [5].

Polymethylmethacrylate (PMMA)-based cement augmentation is commonly employed to improve screw holding in osteoporotic vertebrae. Biomechanical and clinical studies have shown that cement augmentation improves pull-out strength, construct stiffness, and cyclic loading resistance by improving the screw-bone interface [3,6]. Fenestrated pedicle screws have further advanced this concept by allowing controlled cement delivery through radial side ports along the threaded shaft, enabling more uniform cement distribution while reducing injection pressure compared with solid screw augmentation techniques [7,8]. Several studies have suggested that cement distribution pattern and delivery technique may influence fixation performance as much as or more than absolute cement volume [9]. In parallel, clinical series and review articles have supported cement-augmented pedicle screw fixation as a useful strategy in osteoporotic spines, particularly when standard fixation is inadequate [10,11]. Despite these advantages, cement augmentation introduces important clinical trade-offs. Increasing cement volume may improve fixation strength but is associated with a higher risk of cement leakage, which can lead to neurological compression, vascular injury, pulmonary embolism, and thermal damage to surrounding tissues [12-15]. Consequently, there is growing interest in identifying the minimum effective cement volume that provides adequate biomechanical reinforcement while minimizing complication risk.

At the same time, contemporary minimally invasive spinal fixation procedures have started to favor the use of the Jamshidi needle-guided method for the preparation of the pedicle tract and the guidewire insertion [16]. Compared with the conventional method of pedicle probing, the use of the Jamshidi method may alter the compaction of cancellous bone and the tract itself, thus influencing the containment of the cement during the spinal augmentation process [17]. However, limited biomechanical data exist evaluating low-volume cement augmentation strategies, specifically within this minimally invasive insertion paradigm.

Therefore, the primary objective of this study was to evaluate the biomechanical effects of low-volume cement augmentation using fenestrated pedicle screws inserted via a Jamshidi needle technique in a controlled osteoporotic cancellous bone surrogate model. The influence of cement volume (0.5 mL versus 1.0 mL) and delivery technique (prefill versus cannulated injection) on pull-out strength, axial stiffness, and displacement to failure was systematically assessed. It was hypothesized that cement augmentation would improve fixation strength compared with non-augmented constructs, that higher cement volume would produce incremental mechanical benefits, and that cannulated cement injection would provide superior fixation compared with prefill augmentation, particularly at low cement volumes.

## Materials and methods

Study design

This was an in vitro biomechanical comparative study, designed to evaluate the effects of cement volume and cement delivery technique on the fixation strength of fenestrated pedicle screws in an osteoporotic cancellous bone model.

Osteoporotic cancellous bone model

**Figure 1:**
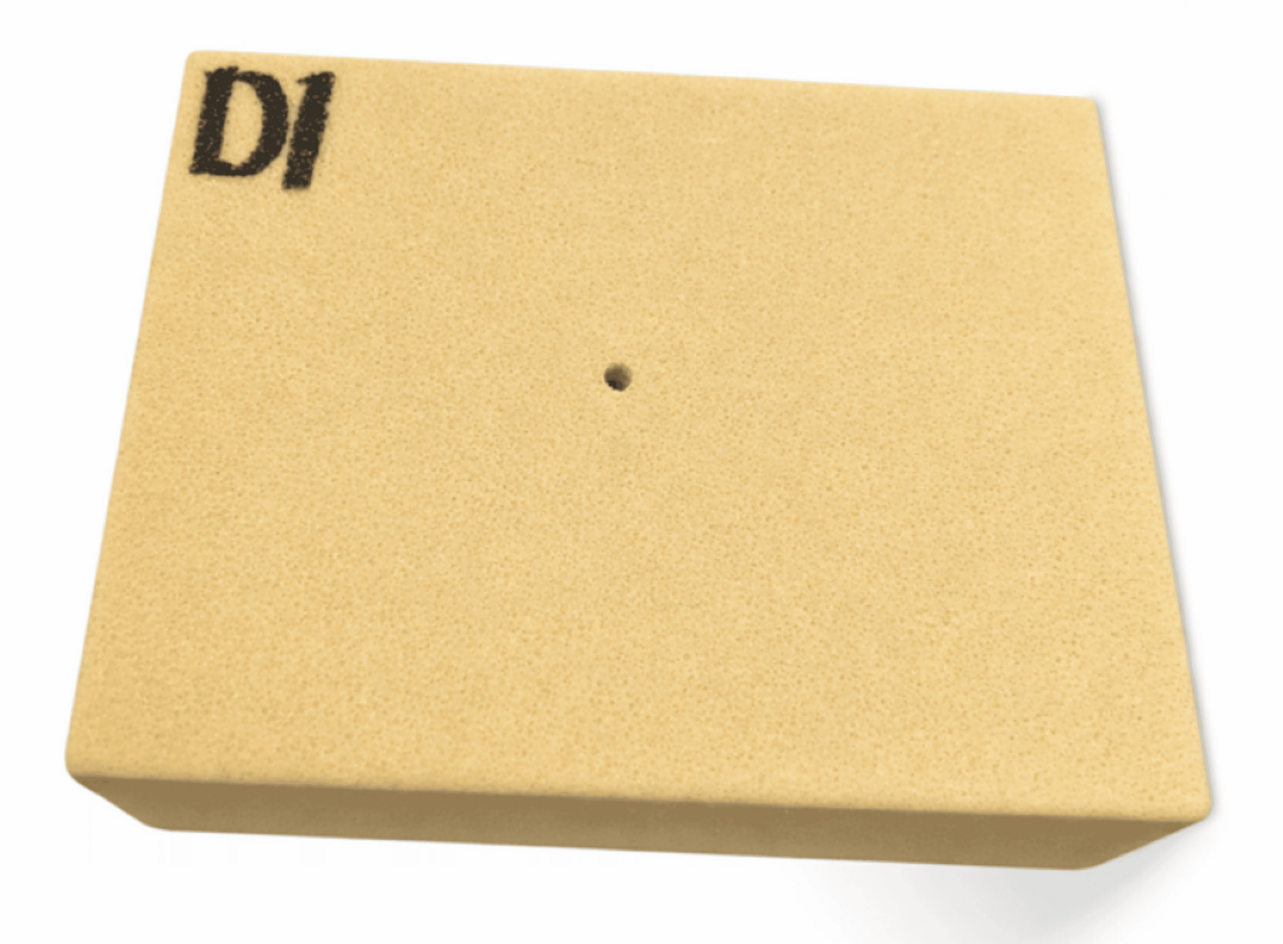
Standardized polyurethane foam block A centrally located pilot hole was prepared using a Jamshidi needle to ensure a uniform screw trajectory. Specimen labeling was performed to maintain group allocation during biomechanical pull-out testing. Image credit: Authors’ own image.

Rigid polyurethane foam blocks (Model #1522-01, Pacific Research Laboratory Inc., Vashon Island, WA, USA) were used in this study as a standardized surrogate for osteoporotic cancellous bone (Figure 1). Grade 10 polyurethane foam (density 0.16 g/cm³), corresponding to severely osteoporotic cancellous vertebral bone, was selected to minimize specimen-to-specimen variability inherent in cadaveric models [5,18]. Rigid polyurethane foam is widely accepted as a standardized material for orthopedic implant testing under American Society for Testing and Materials (ASTM F1839) specifications [19].

**Figure 2:**
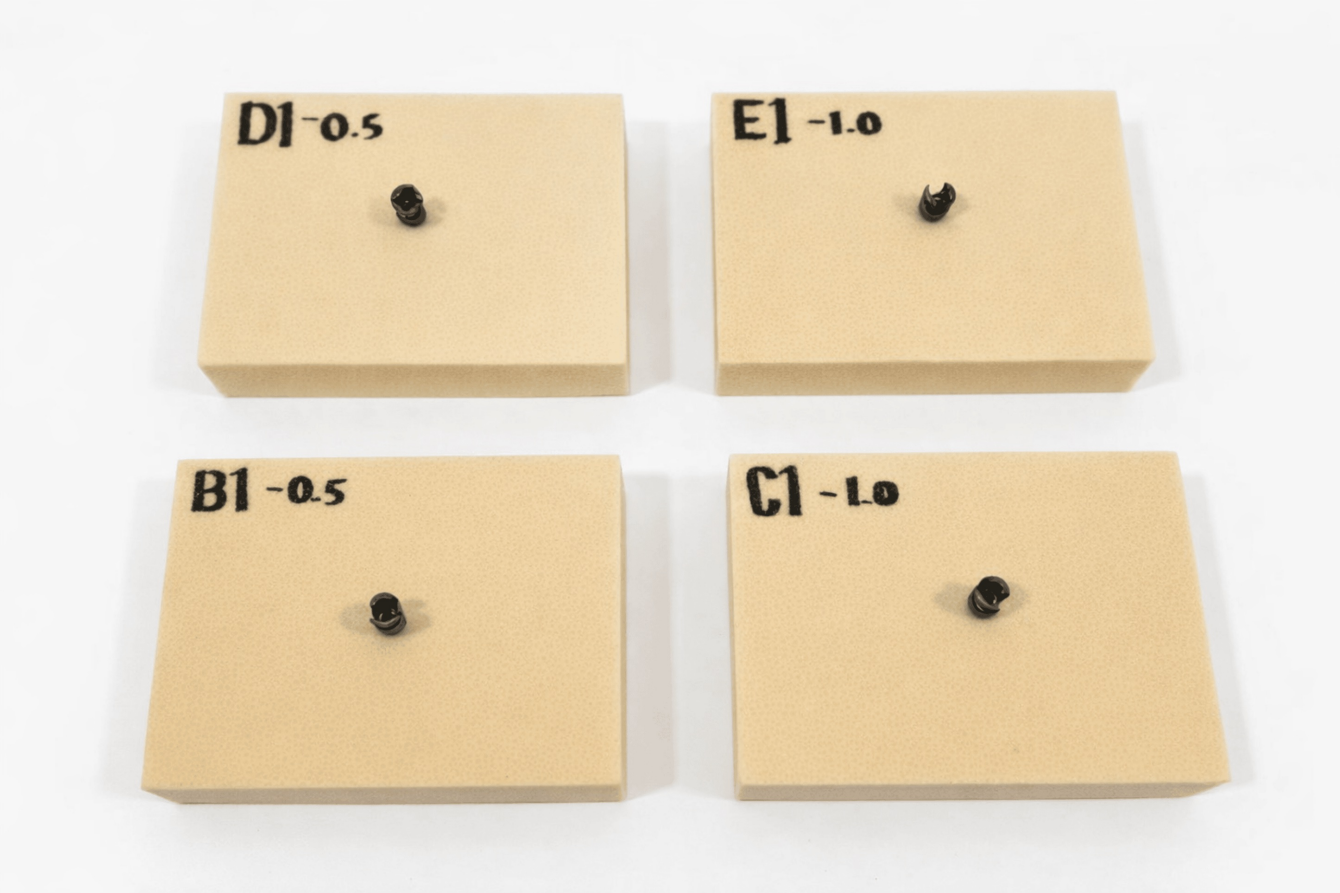
Polyurethane foam blocks with fenestrated pedicle screws before cement augmentation Representative specimens showing fenestrated pedicle screws inserted into osteoporotic cancellous bone surrogate blocks. Image credit: Authors’ own image.

All foam blocks were cut into uniform rectangular specimens, with sufficient dimensions to permit full pedicle screw insertion while maintaining adequate distance between the screw trajectory and the specimen edges to prevent boundary effects during axial pull-out testing (Figure 2).

Pedicle screw system and instrumentation

**Figure 3:**
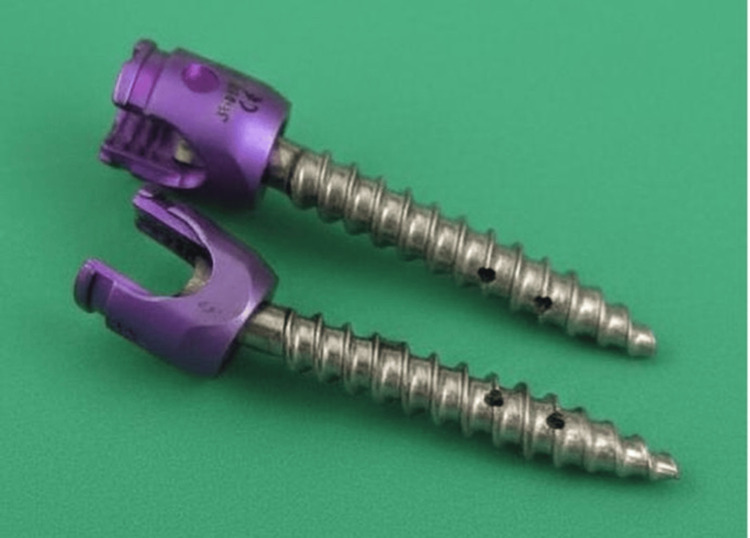
Fenestrated cannulated pedicle screw (6.5 × 40 mm) The screw contains radial fenestrations along the threaded shaft that allow cement extrusion. Image credit: Authors’ own image.

Fenestrated cannulated pedicle screws with a diameter of 6.5 mm and a length of 40 mm (titanium alloy Ti-6Al-4V) were used for all experimental groups (Figure 3). The screws contained multiple radial fenestrations along the threaded shaft to allow controlled cement extrusion through the central cannulation.

**Figure 4:**
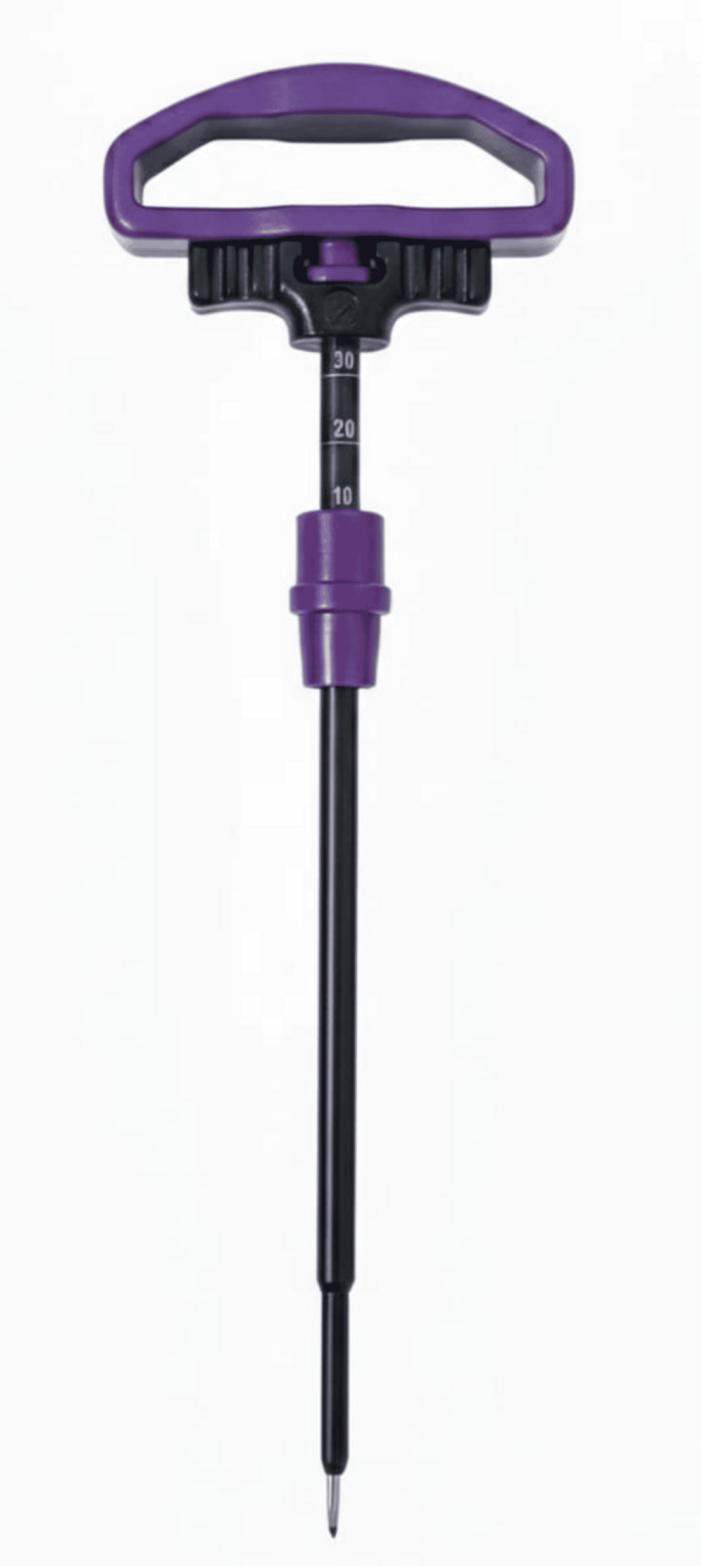
Jamshidi needle used for pedicle tract preparation The Jamshidi needle was used to create the pedicle tract and guidewire pathway prior to fenestrated pedicle screw insertion. Image credit: Authors’ own image.

Pedicle tract preparation was performed using a Jamshidi needle (Figure 4) and the guidewire technique to replicate contemporary minimally invasive percutaneous spinal fixation workflows. Following guidewire placement, the pedicle screws were advanced over the guidewire to a standardized insertion depth of 40 mm, ensuring full threaded engagement within the foam block. All screws were inserted by the same operator using a constant insertion speed of three revolutions per minute to minimize technique-related variability.

Cement material and augmentation protocols

PMMA bone cement (OSTEOPAL® V, Heraeus Medical, Wehrheim, Germany), a radiopaque low-viscosity PMMA cement, was prepared according to the manufacturer’s instructions and used for augmentation. Cement handling and delivery for both injection and prefill techniques were performed during the working phase according to the manufacturer’s recommendations to ensure consistent viscosity during augmentation. Five experimental groups were defined in Table 1.

**Table 1:** Definition of experimental groups and cement augmentation techniques

Group	Augmentation technique
Group A (control)	No cement augmentation
Group B	0.5 mL cement injected through the cannulated screw
Group C	1.0 mL cement injected through the cannulated screw
Group D	0.5 mL cement prefilled into the tract prior to screw insertion
Group E	1.0 mL cement prefilled into the tract prior to screw insertion

For cannulated injection groups, cement was injected through the central lumen of the screw using a syringe until the predetermined volume was delivered. Cement delivery was performed manually through the syringe to achieve the intended augmentation volume. For prefill groups, cement was introduced into the prepared tract before screw insertion, after which the screw was advanced to the final depth.

Following cement delivery, all specimens were allowed to cure under standardized conditions for complete polymerization prior to mechanical testing.

Mechanical testing setup

**Figure 5:**
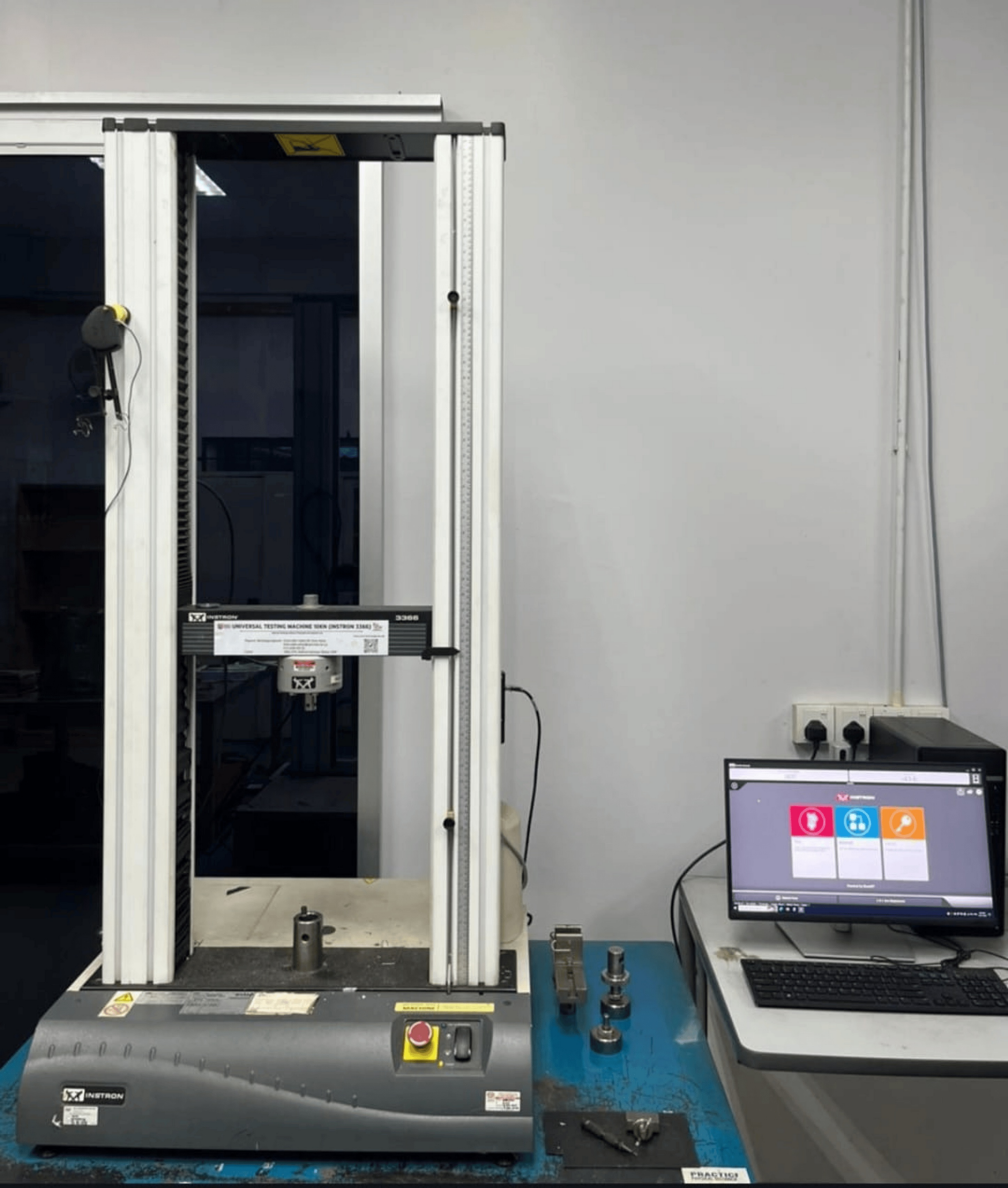
Universal testing machine and computerized data acquisition system for axial pull-out testing Axial pull-out testing was performed using a universal testing machine (Instron 3366, Instron Corp., Norwood, MA, USA) equipped with a 10 kN load cell. The system was operated via a dedicated computer workstation that controlled crosshead displacement and recorded real-time force-displacement data, which were used to derive pull-out strength, axial stiffness, and displacement to failure. Image credit: Authors’ own image.

**Figure 6:**
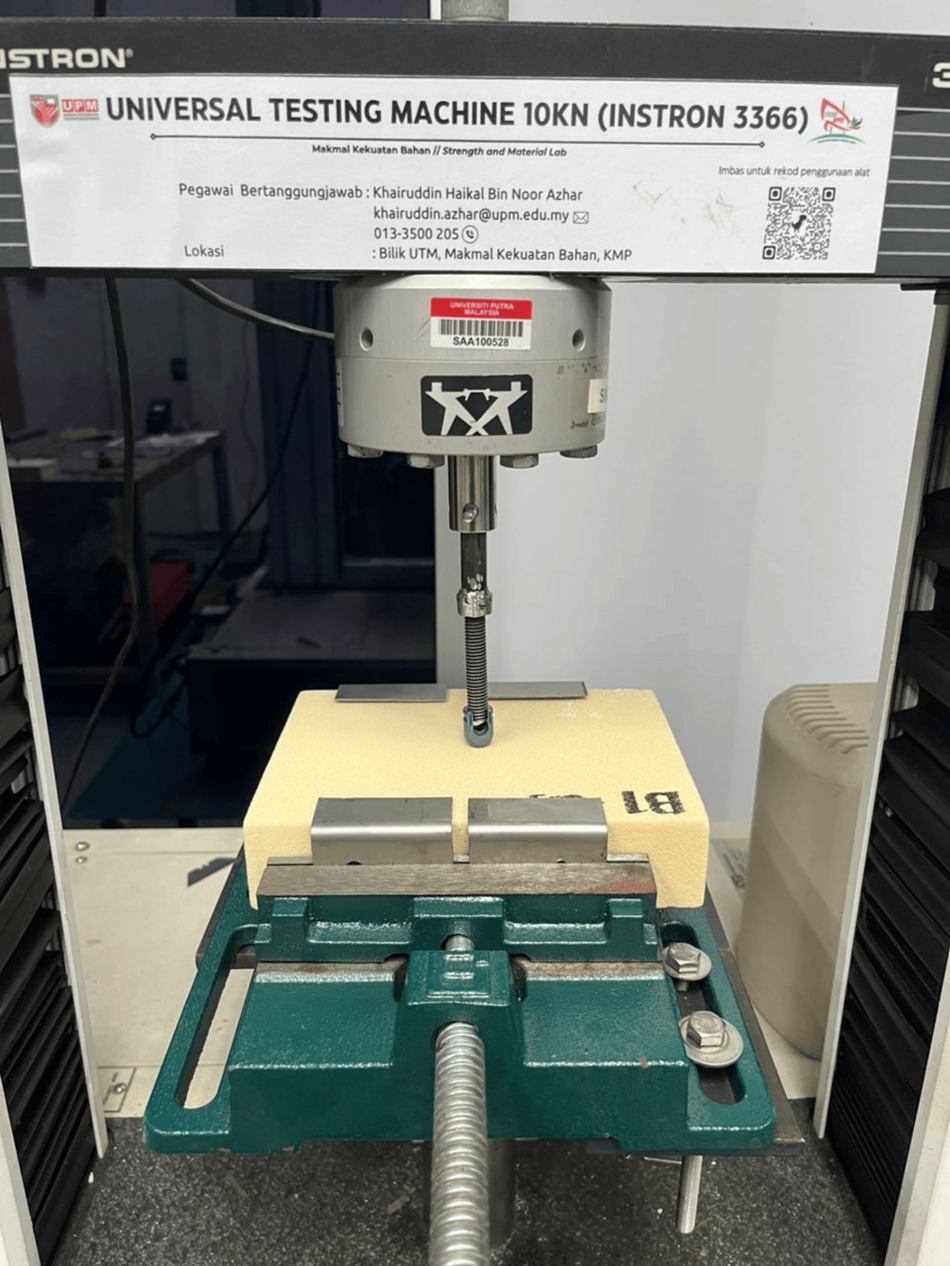
Axial pull-out test setup The pedicle screw was aligned with the loading axis and subjected to axial tensile. Image credit: Authors’ own image.

Axial pull-out testing was performed using a universal testing machine (Instron 3366, Instron Corp., Norwood, MA, USA) equipped with a 10 kN load cell (Figure 5). The screw-foam construct was mounted onto a customized alignment jig to ensure coaxial loading along the longitudinal axis of the screw (Figure 6). Testing was conducted in accordance with ASTM F543 standards for metallic medical bone screws.

**Figure 7:**
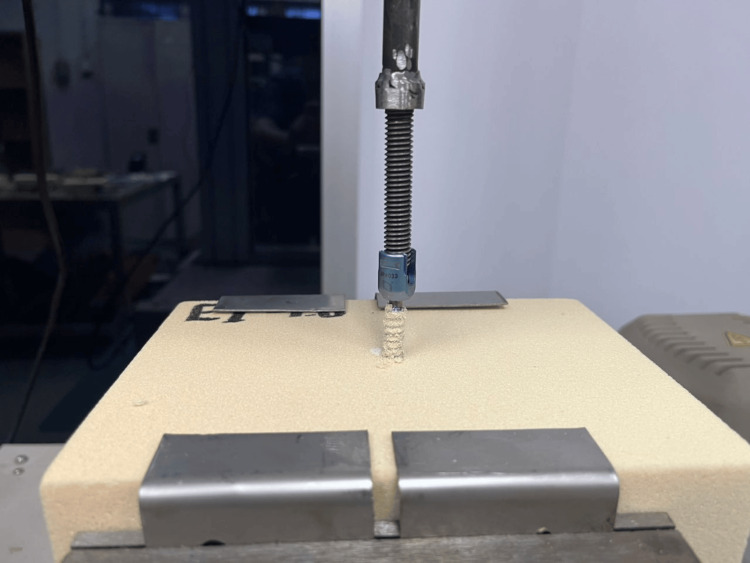
Axial pull out test until failure using the Instron 3366 testing system Image credit: Authors’ own image.

Axial tensile loading was applied at a constant crosshead displacement rate of 5 mm/min until mechanical failure. Failure was defined as the maximum load achieved before complete screw extraction from the foam block. Force-displacement data were continuously recorded at a sampling frequency of 50 Hz using the Instron data acquisition software. A representative pull-out testing configuration during axial loading to failure is shown in Figure 7.

Outcome measures

The following biomechanical parameters were derived from the recorded force-displacement curves: (i) Pull-out strength (N), the maximum axial force recorded prior to failure; (ii) Axial stiffness (N/mm), the slope of the linear elastic region of the force-displacement curve; (iii) Displacement to failure (mm), the axial displacement corresponding to peak load. All measurements were extracted and analyzed using standardized procedures.

Statistical analysis

Statistical analysis was performed using IBM SPSS Statistics for Windows, Version 27 (Released 2019; IBM Corp., Armonk, NY, USA). Continuous biomechanical outcome variables, including pull-out strength (N), axial stiffness (N/mm), and displacement to failure (mm), were expressed as mean ± standard deviation. Given the small sample size (n = 3 per group), non-parametric statistical methods were selected, and overall group comparisons were conducted using the Kruskal-Wallis H test as a conservative alternative to one-way ANOVA. Where statistically significant differences were identified, pairwise post-hoc comparisons were performed using Dunn’s test with Bonferroni correction to control for multiple comparisons. Exact p-values were computed using rank-based analysis, accounting for tie correction and small-sample adjustment. Statistical significance was defined as an adjusted p-value < 0.05 at the 95% confidence level.

## Results

The experimental investigations evaluated the biomechanical effects of varying cement volumes and delivery techniques on the pull-out strength, axial stiffness, and displacement to failure of fenestrated pedicle screws within an osteoporotic cancellous bone model. Statistical analysis, using the Kruskal-Wallis test with Dunn’s post-hoc comparison and Bonferroni correction, identified overall significant differences among experimental groups for all evaluated parameters, while selected pairwise comparisons demonstrated distinct biomechanical performance trends.

Pull-out strength

Mean pull-out strength values for all experimental groups are summarized in Table 2. Kruskal-Wallis analysis demonstrated a statistically significant difference among the five groups (H(4) = 13.50, p = 0.009), indicating that the overall comparison reached statistical significance at the 95% confidence level. Dunn’s post-hoc test with Bonferroni correction was subsequently performed to identify the specific group differences.

**Table 2:** Pull-out strength across experimental groups Values are presented as mean ± standard deviation. Overall comparison: Kruskal-Wallis test, H(4) = 13.50, p = 0.009.

Group	Cement technique	Cement volume (mL)	Pull-out strength (N), mean ± SD	% change vs control
Group A	No cement	0	524.42 ± 12.40	Reference
Group B	Cannulated injection	0.5	874.05 ± 17.41	+66.7%
Group C	Cannulated injection	1.0	1030.32 ± 37.05	+96.5%
Group D	Prefill	0.5	595.02 ± 41.74	+13.5%
Group E	Prefill	1.0	760.08 ± 39.37	+44.9%

Detailed post-hoc pairwise comparisons are presented in Table 3. Dunn’s test with Bonferroni correction demonstrated a statistically significant increase in pull-out strength in Group C compared with Group A (Z = -3.29, adjusted p = 0.010). No other pairwise comparisons reached statistical significance. Although not statistically significant, injection groups demonstrated higher mean pull-out strength values compared with corresponding prefill groups.

**Table 3:** Dunn post-hoc pairwise comparisons for pull-out strength (Bonferroni-adjusted) Bold values indicate statistically significant pairwise comparisons, following Dunn’s post-hoc test with Bonferroni correction (adjusted p < 0.05).

Comparison	Z value	Adjusted p
A vs B	-2.46	0.137
A vs C	-3.29	0.010
A vs D	-0.82	1.000
A vs E	-1.64	1.000
B vs C	-0.82	1.000
B vs D	1.64	1.000
B vs E	0.82	1.000
C vs D	2.46	0.137
C vs E	1.64	1.000
D vs E	-0.82	1.000

Axial stiffness

Axial stiffness values for all experimental groups are summarized in Table 4. Kruskal-Wallis analysis demonstrated a statistically significant difference among the five groups (H(4) = 13.50, p = 0.009), reaching statistical significance at the 95% confidence level, and indicating that at least one group differed significantly from the others. Dunn’s post-hoc test with Bonferroni correction was subsequently performed to identify specific pairwise differences.

**Table 4:** Axial stiffness across experimental groups Values are presented as mean ± standard deviation. Overall comparison: Kruskal-Wallis test, H(4) = 13.50, p = 0.009.

Group	Cement technique	Cement volume (mL)	Axial stiffness (N/mm), mean ± SD	% change vs control
Group A	No cement	0	230.35 ± 1.54	Reference
Group B	Cannulated injection	0.5	324.15 ± 5.57	+40.8%
Group C	Cannulated injection	1.0	336.10 ± 6.04	+45.9%
Group D	Prefill	0.5	268.22 ± 7.64	+16.4%
Group E	Prefill	1.0	303.55 ± 12.68	+31.7%

Detailed post-hoc pairwise comparisons are presented in Table 5. Post-hoc analysis revealed significantly greater axial stiffness in Group C compared with Group A (Z = -3.29, adjusted p = 0.010). No other pairwise comparisons reached statistical significance.

**Table 5:** Dunn post-hoc pairwise comparisons for axial stiffness (Bonferroni-adjusted) Bold values indicate statistically significant pairwise comparisons, following Dunn’s post-hoc test with Bonferroni correction (adjusted p < 0.05).

Comparison	Z value	Adjusted p
A vs B	-2.46	0.137
A vs C	-3.29	0.010
A vs D	-0.82	1.000
A vs E	-1.64	1.000
B vs C	-0.82	1.000
B vs D	1.64	1.000
B vs E	0.82	1.000
C vs D	2.46	0.137
C vs E	1.64	1.000
D vs E	-0.82	1.000

Although not statistically significant, injection groups demonstrated higher mean axial stiffness values compared with corresponding prefill groups.

Displacement to failure

Displacement to failure values for all experimental groups are summarized in Table 6. Kruskal-Wallis analysis demonstrated a statistically significant difference among the five groups (H(4) = 13.03, p = 0.011), reaching statistical significance at the 95% confidence level, and indicating that at least one group differed significantly from the others. Dunn’s post-hoc test with Bonferroni correction was subsequently performed to identify specific pairwise differences.

**Table 6:** Displacement to failure across experimental groups Values are presented as mean ± standard deviation. Overall comparison: Kruskal-Wallis test, H(4) = 13.03, p = 0.011.

Group	Cement technique	Cement volume (mL)	Displacement to failure (mm), mean ± SD
Group A	No cement	0	2.28 ± 0.06
Group B	Cannulated injection	0.5	2.70 ± 0.05
Group C	Cannulated injection	1.0	3.07 ± 0.14
Group D	Prefill	0.5	2.22 ± 0.09
Group E	Prefill	1.0	2.50 ± 0.03

Detailed post-hoc pairwise comparisons are presented in Table 7. Post-hoc analysis revealed significantly greater displacement to failure in Group C compared with Group D (Z = 3.10, adjusted p = 0.019). No other pairwise comparisons reached statistical significance.

**Table 7:** Dunn post-hoc pairwise comparisons for displacement to failure (Bonferroni-adjusted) Bold values indicate statistically significant pairwise comparisons, following Dunn’s post-hoc test with Bonferroni correction (adjusted p < 0.05).

Comparison	Z value	Adjusted p
A vs B	-1.83	0.679
A vs C	-2.65	0.081
A vs D	0.46	1.000
A vs E	-1.00	1.000
B vs C	-0.82	1.000
B vs D	2.28	0.225
B vs E	0.82	1.000
C vs D	3.10	0.019
C vs E	1.64	1.000
D vs E	-1.46	1.000

Although not statistically significant, injection groups demonstrated higher mean displacement values compared with corresponding prefill groups.

## Discussion

In this biomechanical study, we evaluated the effects of cement volume and delivery technique on fixation stability of fenestrated pedicle screws inserted into an osteoporotic surrogate bone model using a Jamshidi needle-guided approach. The principal finding was that cement delivery technique exerted a greater influence on fixation stability than low-volume cement augmentation alone. Cannulated cement injection demonstrated higher mean pull-out strength, axial stiffness, and displacement to failure compared with prefill augmentation, with selected pairwise comparisons reaching statistical significance. These findings are consistent with our study hypothesis and provide clinically relevant insight into low-volume cement augmentation strategies.

Effect of cement augmentation on fixation performance

Cement augmentation resulted in considerable improvement in screw fixation compared with non-augmented constructs. The results demonstrated substantial increases in pull-out strength with injection-based augmentation, with statistically significant improvement observed for the 1.0 mL injection group relative to the control. These findings are consistent with previous studies reporting that PMMA augmentation enhances pedicle screw anchorage in osteoporotic bone by improving the screw-bone interface and load-transfer characteristics [3,6,9].

Osteoporotic cancellous bone is characterized by reduced trabecular connectivity and decreased compressive strength, resulting in premature micromotion and progressive screw loosening under physiological loading conditions [5]. Cement augmentation addresses these limitations by increasing the effective composite interface between the implant and surrounding bone, thereby improving resistance to axial extraction and enhancing construct stiffness. The improvements observed in the present study support the biomechanical advantage of cement augmentation, even at low injection volumes.

Delivery technique as the predominant determining factor for fixations

One of the most clinically relevant findings of this study was that the 0.5 mL cannulated cement injection group demonstrated greater mean pull-out strength than the 1.0 mL prefill augmentation group. Despite using half the cement volume, the injection technique achieved superior mechanical performance compared with the prefill method, highlighting the importance of delivery strategy over absolute cement quantity. This observation is supported by prior biomechanical studies demonstrating that cement distribution pattern and thread-cement interaction are critical determinants of screw holding strength [6-8]. Cannulated fenestrated screws permit radial cement extrusion along the threaded shaft, creating a circumferential cement mantle that interdigitates with surrounding trabecular bone and reinforces the mechanically critical thread-bone interface [6,9]. More uniform cement distribution may alter the failure mechanism by enhancing resistance to trabecular shear and interface disruption.

In contrast, prefill augmentation is susceptible to cement displacement during screw advancement. Cement may redistribute proximally, migrate away from the threaded region, or become compacted within localized regions, particularly at lower cement volumes [3,18]. Such displacement likely contributes to the comparatively reduced and more variable fixation observed in the prefill groups.

Subthreshold behavior of low-volume prefill augmentation

The absence of a statistically significant difference between the 0.5 mL prefill group and the control group suggests an augmentation effect below the threshold of statistical significance rather than experimental failure. Previous studies have reported that such low cement volumes are likely inadequate to create a continuous reinforcing mantle around the threaded region because of the disruption of cement distribution during screw advancement [3,8,18].

Under these conditions, the failure mechanism is likely governed primarily by trabecular shear at the screw-bone interface, resulting in mechanical behavior comparable to that of non-augmented constructs. These findings indicate that low cement volume is not inherently ineffective but becomes biomechanically insufficient when the delivery technique fails to engage the load-bearing region surrounding the threads.

Effects of cement volume, diminishing returns, and leakage risk

Within each delivery technique, increasing cement volume from 0.5 mL to 1.0 mL produced incremental improvements in fixation strength. However, the magnitude of improvement differed substantially between techniques. Cannulated injection demonstrated consistent gains in pull-out strength and displacement to failure, whereas prefill augmentation showed smaller improvements that remained inferior to injection-based augmentation.

Importantly, increasing cement volume is associated with increased risk of cement leakage, including neurological compression, vascular injury, pulmonary embolism, and thermal tissue damage [12-14]. Multiple clinical and biomechanical studies have demonstrated that leakage risk increases with larger cement volumes and uncontrolled cement dispersion [13-15]. Consequently, there is increasing emphasis on identifying the minimum effective cement volume that achieves sufficient fixation while minimizing complication risk.

The deliberate selection of 0.5 mL and 1.0 mL cement volumes in the present study reflects a low-dose augmentation strategy aimed at balancing mechanical efficacy with safety considerations. The finding that 0.5 mL cannulated injection outperformed 1.0 mL prefill supports a technique-first approach in which optimized cement delivery may compensate for reduced cement volume and potentially lower leakage risk.

Influence of Jamshidi needle tract preparation

All screws were placed using a Jamshidi needle and guidewire technique, which is standard in modern minimally invasive spinal fixation procedures. Jamshidi-prepared tracts are typically narrower and may compact cancellous bone differently compared with traditional pedicle probing techniques [16]. This altered tract geometry may enhance cement containment and facilitate controlled radial distribution during cannulated injection, contributing to the superior fixation performance observed in injection groups.

Conversely, these tract characteristics may also contribute to increased cement displacement during prefill augmentation, particularly when low cement volumes are used, thereby reducing effective cement engagement at the thread-bone interface. These observations highlight the importance of evaluating cement augmentation within the context of modern minimally invasive fixation techniques rather than traditional open surgical approaches [16,17].

Interpretation of stiffness and displacement behavior

The increased axial stiffness observed in the cannulated injection groups may indicate improved construct stability during the early postoperative phase. Greater displacement to failure suggests enhanced energy absorption capacity of the augmented construct, thereby delaying catastrophic failure and remaining consistent with the formation of a reinforced cement-bone interface.

In contrast, stiffness and displacement values in the prefill augmentation groups did not differ substantially from those of the non-augmented groups. This observation suggests that limited cement engagement at the thread-bone interface and possible cement displacement during screw insertion may reduce the mechanical contribution of prefill augmentation, particularly at low cement volumes. Consequently, effective cement distribution, rather than cement presence alone, appears to be a key determinant of construct stability.

Hypothesis-oriented summary of findings

The biomechanical trends observed in this study support the primary hypothesis. Cement augmentation improved fixation compared with non-augmented constructs, with the 1.0 mL cannulated injection group demonstrating approximately a 96% increase in pull-out strength and a 45%-46% increase in axial stiffness relative to control.

Furthermore, cement delivery technique exerted a greater influence on fixation performance than cement volume within the low-dose range evaluated. The 0.5 mL cannulated injection group outperformed the 1.0 mL prefill group despite using half the cement volume, suggesting that circumferential cement distribution through the fenestrated screw enhances thread-bone interdigitation more effectively than prefill augmentation.

Given the limited sample size (n = 3 per group), these findings should be interpreted as consistent biomechanical trends rather than definitive statistical conclusions.

Summary of mechanical performance

Across all biomechanical outcome measures, a consistent pattern of fixation performance was observed. Cannulated cement injection demonstrated the greatest improvements in pull-out strength, axial stiffness, and displacement to failure, followed by higher-volume prefill augmentation. Low-volume prefill augmentation provided minimal mechanical benefit and did not differ substantially from non-augmented fixation in key biomechanical comparisons. The overall fixation performance can be ranked as follows in Table 8.

**Table 8:** Overall ranking of fixation performance across augmentation techniques Ranking is based on comparative performance across pull-out strength, axial stiffness, and displacement to failure.

Ranking order	Description
1.	1.0 mL injection
2.	0.5 mL injection
3.	1.0 mL prefill
4.	0.5 mL prefill
5.	No cement

This pattern suggests that cement delivery technique exerts a greater biomechanical influence than low-dose cement volume, particularly within the 0.5-1.0 mL range evaluated in this study.

Clinical implications 

From a clinical perspective, these findings are particularly relevant in patients with osteoporotic bone, where balancing adequate implant fixation strength with minimizing cement-related complications is critical. The effectiveness of low-volume cannulated injection in providing improved fixation compared with higher-volume prefill augmentation suggests that this technique may reduce cement usage while maintaining mechanical stability. Previous biomechanical modeling studies have similarly demonstrated that even relatively low cement volumes can substantially restore construct stiffness when adequate cement distribution is achieved [18]. These findings support the adoption of low-dose, technique-driven cement augmentation strategies, particularly in minimally invasive spinal fixation procedures. Such an approach may help reduce cement-related complications while preserving adequate fixation strength in osteoporotic patients. Nevertheless, careful patient selection and meticulous injection technique remain essential to optimize outcomes and minimize procedural risks.

Limitations

This study utilized a standardized synthetic osteoporotic cancellous bone model (0.16 g/cm³ polyurethane foam) in accordance with ASTM specifications. While this approach ensures uniform mechanical comparison and minimizes inter-specimen variability, it does not replicate native pedicle anatomy, cortical shell contribution, trabecular heterogeneity, or biological remodeling processes present in vivo. Consequently, absolute fixation values may differ from those observed in cadaveric or clinical settings.

The sample size was limited to three specimens per group. Although appropriate non-parametric statistical methods were employed and significant intergroup differences were detected, the findings should be interpreted primarily as consistent biomechanical trends rather than definitive statistical generalizations. This study was designed as a pilot biomechanical investigation to evaluate relative performance patterns of low-volume cement augmentation strategies.

Mechanical testing was limited to single-cycle axial pull-out analysis and did not incorporate cyclic loading or fatigue simulation. Additionally, cement leakage and three-dimensional distribution patterns were not directly assessed. Future studies incorporating cyclic loading protocols and imaging-based cement analysis would further clarify long-term construct behavior and clinical translation.

## Conclusions

Cement augmentation led to improved fixation performance of fenestrated pedicle screws in the osteoporotic cancellous bone surrogate model used in this study. The results indicate that the delivery technique had a greater impact on fixation performance than low-dose cement volume. Cannulated cement injection was associated with clear improvements in pull-out strength, axial stiffness, and displacement to failure, even at a volume of 0.5 mL, while low-volume prefill augmentation showed limited mechanical benefit and greater variability.

Taken together, these findings suggest that a technique-driven approach to cement augmentation may allow effective fixation to be achieved while keeping cement volumes low and reducing the risk of related complications. Within current minimally invasive spinal fixation practice, low-volume cannulated injection appears to be a biomechanically efficient option for improving screw anchorage in osteoporotic bone. Further work incorporating cyclic loading protocols and direct assessment of cement leakage will be important to confirm these observations under more clinically representative conditions.
